# Sarcoidosis Treatment with Antifungal Medication: A Follow-Up

**DOI:** 10.1155/2014/739673

**Published:** 2014-12-04

**Authors:** Marjeta Terčelj, Barbara Salobir, Mirjana Zupancic, Ragnar Rylander

**Affiliations:** ^1^Clinic of Pulmonary Diseases and Allergy, University Medical Centre, Zaloska 7, 1000 Ljubljana, Slovenia; ^2^Laboratory Department, Children's Hospital, University Medical Center, 1000 Ljubljana, Slovenia; ^3^BioFact Environmental Health Research Center, 44391 Lerum, Sweden

## Abstract

*Introduction*. The aim of the study was to compare treatment of sarcoidosis with antifungal or corticosteroid medication.* Methods.* In patients with sarcoidosis antifungal medication (*n* = 29), corticosteroids (*n* = 21) or a combination (*n* = 27) was given. Nine patients allotted to antifungal medication were later given corticosteroids because of the lack of regression of the disease. X-ray scores for the severity of granuloma infiltration were determined. Chitotriosidase and angiotensin converting enzyme were determined. The time in months till remission was observed as well as the number of recurrences.

## 1. Introduction

Sarcoidosis is an inflammatory, granulomatous disease present in populations worldwide and with a higher incidence in some ethnic groups [1, 2]. The conventional treatment is with corticosteroids. During recent years there have been an increasing number of studies reporting a connection between sarcoidosis and environmental exposure to fungi [3–5]. Fungal exposure at home was higher among patients with sarcoidosis as compared to controls and highest among those with recurrence of the disease. *β*-Glucan, a major constituent of the fungal cell wall, has been determined in bronchoalveolar lavage (BAL) fluid in patients with sarcoidosis and controls [6]. The amounts were significantly higher among patients with sarcoidosis and were related to the fungal exposure at home as well as to serum levels of IL-2R and IL-12.

These studies showing a relation between exposure to fungi and different experimental and clinical effects have prompted studies where antifungal medication has been given, together with or without simultaneous corticosteroid treatment. In a first study, 18 patients with chronic sarcoidosis were given antifungal medication in addition to the usual corticosteroid treatment. 15 of these patients improved after 6 months [7]. In a subsequent study 39 newly diagnosed patients received corticosteroids for 6 months and 18 received antifungal medication [8]. Pulmonary granuloma infiltration was significantly less severe in the antifungal group as were the values of angiotensin converting enzyme (ACE) and chitotriosidase (CTO). In a case study on osseous sarcoidosis, treatment with antifungal medication improved the condition [9].

These treatment studies covered a relatively short time period and the time till remission or the risk of recurrence was not studied. Before a final conclusion regarding the efficiency of antifungal treatment can be drawn, further studies are required with a larger number of subjects, followed over a longer time period.

The present study was undertaken to compare the effects of corticosteroid and antifungal treatment with regard to remission and recurrence of the disease after termination of treatment.

## 2. Material and Methods

Patients with sarcoidosis attend the Clinic of Respiratory Diseases and Allergy at the University Medical Centre, Ljubljana, Slovenia. The diagnosis of sarcoidosis at the clinic adheres to the ATS criteria [10] and comprises the following steps. Bronchoscopy is made and 5 to 10 transbronchial biopsies of lung parenchyma and lymph nodes are taken. BAL is performed with 200 mL saline to determine CD8+ cells and the CD4+/CD8+ ratio. The presence of noncaseating granulomas is verified histologically. If a biopsy is not considered representative, the patient undergoes surgical pulmonary or lymph node biopsy. Aspiration is performed from one lobe for culturing fungi and bacteria including tuberculosis. Most biopsies are stained (silver staining, Gomori) to verify the absence of pathogenic fungi.

The subjects in the present study were 77 Caucasian patients with sarcoidosis who had undergone treatment during the years 2006–2011. After a three-month expectation period to avoid including patients with a spontaneous regression, treatment was initiated. Criteria for treatment were symptoms such as fatigue, joint pains, and dyspnoea, X-ray changes indicating granuloma formation, and involvement of organs other than the lung, such as spleen, liver, and eyes at the time for treatment decision.

The patients were given the choice of antifungal (posaconazole 300 mg/day or ketoconazole 200 mg/day) or corticosteroid (methylprednisolone initially 0.4 mg/kg body weight/day for the first month and then 20 mg every second day) medication. The dose of antifungal was low for precautionary reasons and because there was no sign of fungal infection.

A double-blind, placebo design was not possible as all patients needed treatment at the time when medication was chosen. A randomized study was also not possible as the subjects had to be informed about the side effects, and some subjects insisted on antifungal medication, aware of the results from previous studies. The side effects were explained and some patients chose the antifungal and others the corticosteroid treatment. Those who let the physician choose were given antifungal treatment. In summary 21 subjects were given corticosteroid medication (C group) and 56 antifungal medication. Among those with antifungal treatment, corticosteroid was added later in 27 subjects because of increased symptoms of fatigue, joint pains, and shortness of breath (AF + C group). Nine subjects in the antifungal group showed no clinical improvement or had worsening of symptoms or X-rays at 6 months after initiation of treatment. They received an additional treatment with corticosteroids. These patients are reported separately. In summary 20 subjects were thus given antifungal only (AF group).

X-rays were taken and a grading scheme for the presence of granuloma was used as described previously [7, 8]. The X-rays were read by two experienced radiologists, unaware of the status of the patient, grading granuloma infiltration according to a numerical score (0–4) and judging the size and extension of the infiltrates (0 normal, 1 about 25% of lung field involved, 2 up to 50%, 3 up to 75%, and 4 virtually the whole lung field involved). Repeat evaluations on two successive occasions showed only minor deviations in the score classification. Pulmonary function (VC, DLco) was determined and expressed as a percentage of the expected.

Serum samples were taken and ACE was determined using a colorimetric method and expressed as *μ*Kat/L [11]. CTO was determined using 22 *μ*M 4-methylumbelliferyl-*β*-D-N,N′,N′′-triacetylchitotriosiose (Sigma) in citrate phosphate buffer (pH 5.2) and expressed as nmol/h/mL [12].

The disease was monitored using X-ray, and inflammatory cytokines in serum were determined at the time of diagnosis, after about 6 months, and at the end of treatment (remission). Remission comprised absence of symptoms, signs of active disease and ACE, and CTO lower than the initial values. Time till remission in months was determined for all subjects. In addition the number of patients with a recurrence of the disease was determined.

### 2.1. Statistics

Group data were reported as mean and SEM. Differences between groups were evaluated using the Mann-Whitney test and Fisher's exact test. Correlations were evaluated using Spearman's test. A *P* value of ≤0.05 was considered the level of significance.

## 3. Results

Table 1 reports the characteristics of the study subjects at initiation of treatment.

The demographic characteristics were very similar in the different groups except that there were more cases with extrapulmonary involvement in the C group and that DLco was lower in the C group (*P* = 0.008).

The average duration of treatment till remission was 12.1/0.7 months in the AF group, 13.7/0.8 months in the C group (NS), and 17.6/2.2 in the AF + C group which was significant from AF group (*P* = 0.023).

Table 2 reports the clinical parameters at initiation of treatment, after 6 months of treatment, and at remission.

Before treatment the X-ray score was slightly higher, although not statistically significant, in the AF + C group as compared to the AT group and the C group. CTO was significantly lower in the AF group as compared to the C group (*P* = 0.001) and the AF + C group (NS). DLco was lower in the C group as compared to the AF and AF + C groups (*P* = 0.07 and 0.039, resp.).

Over time there was an improvement in X-ray scores in all groups but a larger recovery in the AF group as compared to the C group at remission (*P* = 0.003 and NS, resp.). A similar trend was found for CTO, but here the decrease was most marked in the AF + C group. Also ACE and DLco values improved in all groups with no significant differences between the groups.

At remission the X-ray scores were lower in the AF and AF + C groups as compared to the C group (*P* = 0.015 and NS, resp.). Also the CTO values were lower in the AF group and the AF + C group as compared to the C group (NS and *P* = 0.017, resp.).

**Figure 1:**
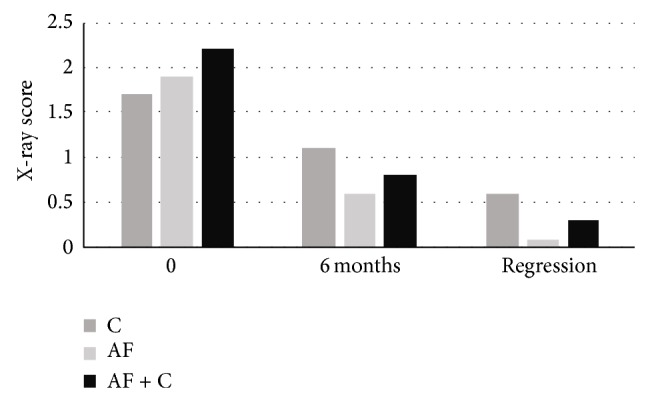
X-ray scores at different times after initiation of treatment with corticosteroid (C, *n* = 21), with antifungal medication (AF, *n* = 20), and with antifungal + corticosteroid (AF + C, *n* = 27).

Figure 1 illustrates the X-ray scores at different times from initiation of treatment.

**Figure 2:**
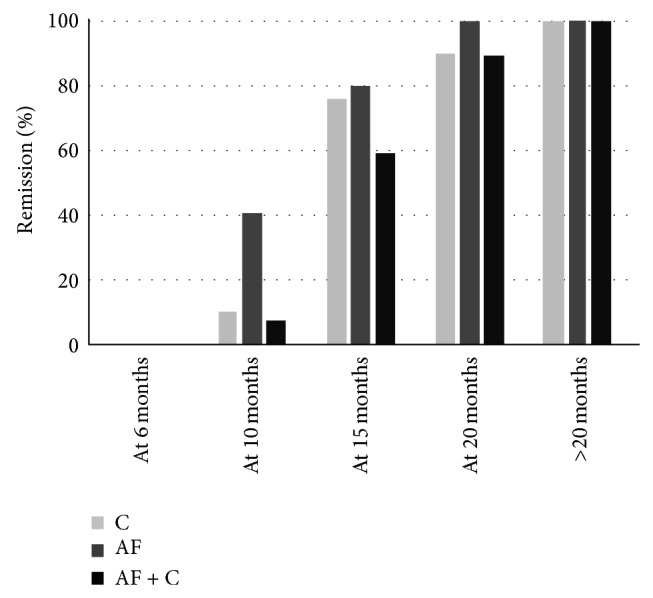
Proportion of patients with remission at different times after initiation of treatment for those treated with corticosteroid (C, *n* = 21), with antifungal medication (AF, *n* = 20), and with antifungal + corticosteroid (AF + C, *n* = 27).

Figure 2 reports the proportion of patients with remission at different times after initiation of treatment in the C, AF, and AF + C groups.

At 10 months a larger proportion of subjects in the AF group had remission as compared to the C and AF + C groups (*P* = 0.033 and NS). At 20 months the proportion with remission in the AF + C group was lower than in the other groups (NS). Among those who took longer than 20 months to remission, there was one subject in the C group (25 months till remission) and five in the AF + C group (24, 26, 28, 50, and 56 months till remission).

Regarding recurrence of sarcoidosis after treatment, there were seven cases in the C group, one in the AF group, and two in the AF + C group (different from C group, *P* = 0.021 and 0.026, resp.).

The disease characteristics were analysed for the nine subjects initially given antifungal treatment, but corticosteroid treatment was added. One subject had developed involvement of the central nervous system, two had sarcoidosis in the eyes, and one subject developed side effects from the antifungal medication. The initial X-ray score in this group was higher than in the C group (2.33/2.2 versus 1.71/0.1, *P* = 0.035). ACE was also higher (0.55/0.7 versus 0.24/0.03, NS). The duration of therapy till remission was 15.3/3.8 months. There were no differences for the other parameters.

## 4. Discussion

The main result from this study was a larger proportion of patients with remission after 6–10 months of treatment, a lower X-ray score at remission, and fewer cases of recurrence among subjects treated with antifungal medication.

The study has some limitations. The number of subjects is small and the study is thus of an exploratory nature. There were, however, marked differences between the treated groups, which were statistically significant. Medication with corticosteroids and antifungal was not distributed randomly or in a double blind fashion. The absence of a randomized distribution of medication could induce a selection of cases with regard to severity at the initiation of treatment. It is also possible that a precaution regarding treatment and the absence of experience in the initial parts of the study could make the responsible physician choose corticosteroids instead of antifungal medication in more severe cases. A comparison between the C and AF groups showed higher values of CTO and lower values of DLco in the C group, suggesting a selection. The AF + C group had a higher X-ray score initially, but it was lower than the C groups at remission. Furthermore the values of CTO were lower and those of DLco were higher in the AF group at remission. In view of this it is unlikely that an initial selection had a major influence on the treatment results in the AF group.

In the AF group corticosteroids had to be added for nine patients due to the clinical development of the disease. These patients could have had a more severe form of the disease (as suggested by the higher X-ray score at start of treatment), resisting the antifungal treatment or a form of sarcoidosis not related to fungal exposure. Another possibility is that they might have had a lower than normal absorption of the antifungal medication. Furthermore the dose for antifungal medication used was below the recommended therapeutical dose because of safety reasons. This might also explain the need for additional treatment in these patients.

The antifungal treatment was efficient although analysis of BAL and lung tissue could not demonstrate the presence of pathogenic fungi. In the environment, however, the major fungal burden is nonpathogenic fungi such as* Penicillium*. Previous studies have reported that fungal exposure at home was higher among patients with sarcoidosis than controls [5]. There is also a relation between fungal exposure at home and the amount of *β*-glucan in BAL of subjects with sarcoidosis [6]. From an environmental exposure point of view the finding that antifungal medication is efficient in sarcoidosis is thus not surprising.

## 5. Conclusion

The results demonstrate that the effect of antifungal medication in newly diagnosed cases of sarcoidosis was similar to that after treatment with corticosteroids in 21 out of 30 patients. At six months after initiation of treatment and at remission, the X-ray changes were less severe among those treated with antifungal medication. The risk of recurrence was also significantly lower in this group. The results suggest that patients should be informed about the possibility to take antifungal medication and that attention should be given to their possible exposure to high levels of fungi. Further studies on patients with severe forms of sarcoidosis and using higher levels of antifungal medication are warranted.

## Figures and Tables

**Table 1:** Characteristics of subjects in different groups at initiation of treatment. Number (*n*) or arithmetic mean/SEM.

Treatment	Corticosteroid	Antifungal	AF + C
*n*	21	20	27
Age years	44.0/3.4	42.4/2.3	41.1/2.0
Smokers *n*	3	1	4
Females *n*	10	9	12
Stage 2 *n*	19	20	21
Stage 3 *n*	2	0	6
Extrapulmonary *n*	12	8	9
CD4/CD8	6.9/0.9	6.4/0.9	6.5/0.8
VC %	89.3/2.9	97.3/1.0	93.7/1.9
FEV_1_ %	89.1/3.0	94.3/2.3	92.5/2.3
DLco %	80.5/3.6	92.3/3.3	89.6/3.5

**Table 2:** Clinical parameters at initiation of treatment, after 6 months of treatment, and at remission. Mean/SEM.

Treatment	Corticosteroid	Antifungal	AF + C
*n*	21	20	27
X-ray score			
Before	1.7/0.1	1.9/0.2	2.2/0.2
6 months	1.1/0.1	0.6/0.2	0.8/0.1
Remission	0.6/0.2	0.1/0.1	0.3/0.1
CTO nmol/h/mL			
Before	843/86	454/70	632/108
6 months	260/44	372/149	184/52
Remission	342/57	271/66	178/37
ACE *µ*Kat/L			
Before	0.44/0.05	0.44/0.04	0.41/0.03
6 months	0.20/0.02	0.38/0.04	0.26/0.03
Remission	0.24/0.03	0.34/0.03	0.24/0.02
DLco % pred.			
Before	80.5/3.6	92.3/3.3	89.6/3.5
6 months	79.0/5.5	92.9/3.7	91.1/4.7
Remission	90.8/3.6	95.7/3.5	93.4/3.6

## Abbreviations

ACE: Angiotensin converting enzyme
CTO: Chitotriosidase
DLco: Pulmonary diffusion capacity
VC: Vital capacity.
